# Nanomedicine: Insights from a Bibliometrics-Based Analysis of Emerging Publishing and Research Trends

**DOI:** 10.3390/medicina55120785

**Published:** 2019-12-15

**Authors:** Nicola Luigi Bragazzi

**Affiliations:** Department of Mathematics and Statistics, York University, Toronto, ON M3J 1P3, Canada; bragazzi@yorku.ca

**Keywords:** nanomedicine, bibliometrics, scientometrics

## Abstract

*Background and Objectives:* Nanomedicine, a term coined by the American engineer Eric Drexler (1955) and Robert Freitas Jr. (1952) in the nineties, can be defined as a complex, multi-disciplinary branch of medicine, in which nano-technologies, molecular biotechnologies, and other nano-sciences are applied at every step of disease management, from diagnosis (nano-diagnostics) to treatment (nano-therapeutics), prognosis, and monitoring of biological parameters and biomarkers. Nanomedicine is a relatively young discipline, which is increasingly and exponentially growing, characterized by emerging ethical issues and implications. Nanomedicine has branched out in hundreds of different sub-fields. *Materials and Methods:* A bibliometrics-based analysis was applied mining the entire content of PubMed/MEDLINE, using “nanomedicine” as a Medical Subject Heading (MeSH) search term. *Results:* A sample of 6696 articles were extracted from PubMed/MEDLINE and analyzed. Articles had been published in the period from 2003 to 2019, showing an increasing trend throughout the time. Six thematic clusters emerged (first cluster: molecular methods; second cluster: molecular biology and nano-characterization; third cluster: nano-diagnostics and nano-theranostics; fourth cluster: clinical applications, in the sub-fields of nano-oncology, nano-immunology and nano-vaccinology; fifth cluster: clinical applications, in the sub-fields of nano-oncology and nano-infectiology; and sixth cluster: nanodrugs). The countries with the highest percentages of articles in the field of nanomedicine were the North America (38.3%) and Europe (35.1%). *Conclusions:* The present study showed that there is an increasing trend in publishing and performing research in the super-specialty of nanomedicine. Most productive countries were the USA and European countries, with China as an emerging region. Hot topics in the last years were nano-diagnostics and nano-theranostics and clinical applications in the sub-fields of nano-oncology and nano-infectiology.

## 1. Introduction

Nanomedicine can be defined as a complex, multi-disciplinary branch of medicine, in which nano-bio-technologies (including nano-technologies and molecular biotechnologies), and other nano-sciences are applied at every step of disease management, from diagnosis (the so-called nano-diagnostics) to treatment (nano-therapeutics), prognosis, and monitoring of biological parameters and biomarkers [1]. The term “nanomedicine” was coined by the American engineer Eric Drexler (1955) and Robert Freitas Jr. (1952) in the nineties [2], with the publication of the multi-volume textbook entitled “Nanomedicine”, released in October 1999.

Nanomedicine potentially enables physicians to detect a disorder early, before the insurgence of its clinical manifestations and symptoms, as well as to provide drugs in a rational, precise, and targeted way, thereby minimizing the risk of the occurrence of side-effects as much as possible [3].

Furthermore, different super-specialties such as nano-surgery [4,5], nano-neurosurgery [6,7], nano-otorhinolaryngology [8], nano-dentistry [9], nano-ophthalmology [10], nano-neurology [11], nano-cardiology [12], nano-orthopedics [11], nano-infectiology [11], and nano-oncology [13], among others [11], are emerging within nanomedicine.

Nanomedicine is a relatively young discipline, which is increasingly and exponentially growing, characterized by emerging ethical issues and implications [14]. Nanomedicine has branched out in hundreds of different sub-fields [1]. Therefore, the purpose of this investigation was to apply bibliometrics tools to the field of nanomedicine, reflecting its various aspects of nanomedicine (theoretical, applied, translational, regulatory ones) and their spatio-temporal evolution.

In recent years, researchers have been using scientometrics, a branch of information science and a sub-field of bibliometrics, to quantitatively investigate emerging research patterns in the scientific literature [15]. Scientometrics enables also to assess trends in article citations and how these indicators and measurements can have an impact on policy and management. Using scholarly databases and visualization technology allows researchers to gain a good understanding of the of publication trend related to a given topic [15].

To the best of our knowledge, there is a dearth of information concerning publishing and research patterns in the field of nanomedicine. Therefore, the purpose of this study was to conduct a comprehensive analysis of nanomedicine related scientific literature since the nineties.

## 2. Materials and Methods

The present quantitative study was based on medical informatics, data and text mining, and scientometrics techniques [15].

The entire content of PubMed/MEDLINE was searched from January 1990 to 11 November 2019. Concerning inclusion and exclusion criteria, in order to retain only articles strictly relevant to the topic under study, we limited our search to scholarly items dealing with “nanomedicine”, using “nanomedicine [MeSH]” as a keyword.

Search was performed without language restriction. All records relevant to the field of nanomedicine were deemed eligible and, as such, included in the present investigation.

Regarding data extraction, data were downloaded in comma-separated values (CSV) and MEDLINE formats.

Relevant data were extracted: namely, (i) number of documents published within the study period, (ii) the top authors and their co-authorship relations, (iii) the top institutes/research centers with the highest number of documents related to nanomedicine, and (iv) the top countries with the highest number of documents dealing with nanomedicine.

Regarding analysis, an ad hoc visualization software was used to visualize nanomedicine-related research hotspots, patterns, directions of research development and other relevant trends, using networks and graphs. All data were imported and loaded into VOSviewer Version 1.6.13 (freely accessible and downloadable at https://www.vosviewer.com/) [16]. For visualization publication density worldwide (that is to say, publication trends in the different countries), the open-source tool GunnMap was used, as well as Mapping MEDLINE (freely accessible at https://esperr.github.io/mapping-medline/).

## 3. Results

A sample of 6696 articles was extracted from PubMed/MEDLINE and analyzed: 2724 (40.7%) were reviews, and 14 were clinical trials (0.2% of the entire sample).

**Figure 1:**
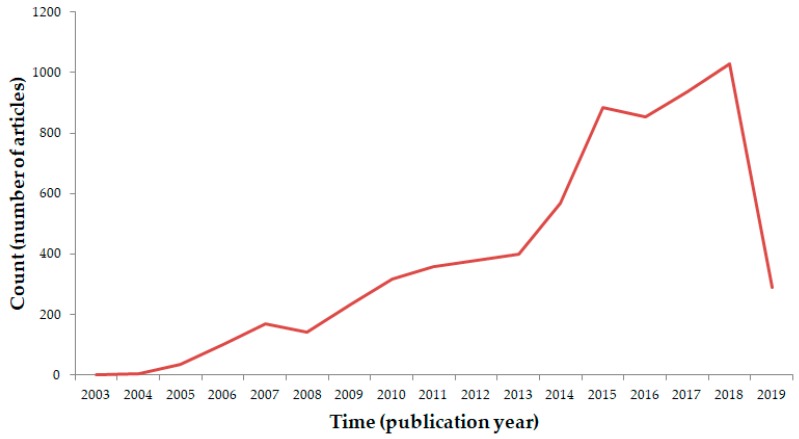
Publishing trend of articles in the field of nanomedicine (2003–2019).

Articles had been published in the period from 2003 to 2019 (Figure 1), showing an increasing trend throughout the time.

**Figure 2:**
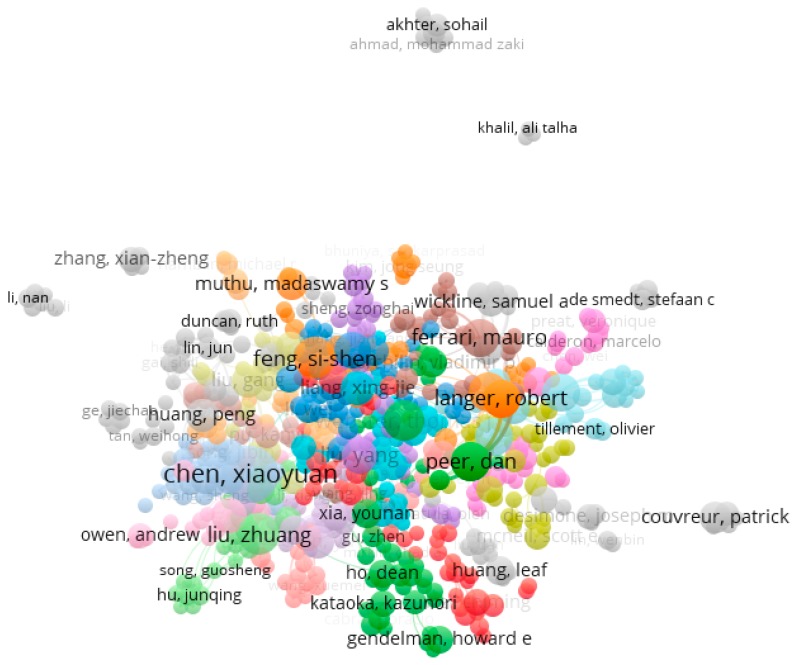
Network of authors publishing and doing research in the field of nanomedicine. Colors represent the authors clusters based on co-authorship.

**Figure 3:**
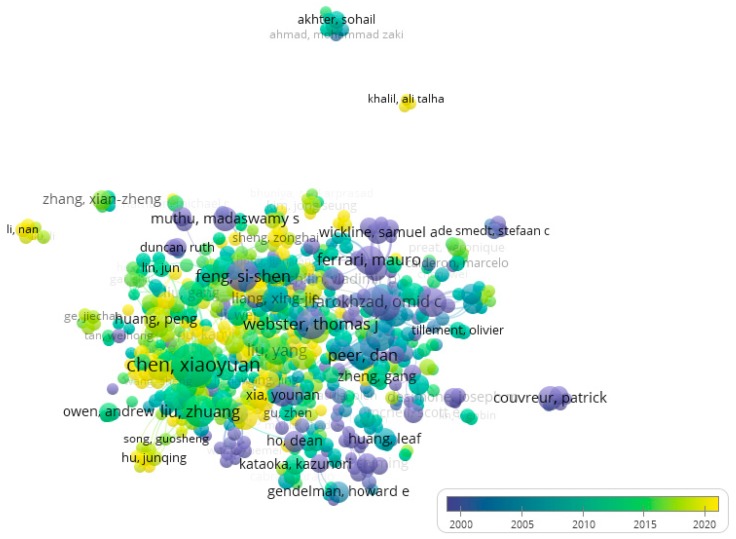
Network of authors publishing and doing research in the field of nanomedicine. Colors represent the authors clusters based on co-authorship and publication year.

Four thousand and twenty-six (60.1%) articles concerned the super-specialty of nano-oncology, whereas 239 (3.6%) the sub-field of nano-nutrition and 132 (2.0%) nano-infectiology. Articles had been written by 25,107 authors. Seven hundred and fourteen (2.8%) had authored more than 5 articles in the field of nanomedicine (median 6 articles, range 5–67). The average link of strength (as indicator of co-authorship) was 12 (range 0–294). Five hundred and ninety-two authors (82.9% of the subset of authors with more than 5 publications, 2.4% of all authors) were highly connected with each other. Based on authors relationships, a network comprising of 2909 links (total link of strength 5927) and 31 clusters was generated (Figure 2). Stratifying the authors network based on time of authorship, it emerged that Chinese authors were more represented in the recent years (Figure 3).

Topics were inferred from a list of 13,125 keywords, 1765 of which (13.4%) co-occurred more than 5 times. From these, the 1000 with the highest link of strength were selected. Six clusters were obtained (number of links 94,431, total link strength 451,670), as shown in Table 1.

Articles had been written by authors belonging to 13,115 organizations/institutions or research centers. Those with more than 5 articles in the field of nanomedicine are reported in Supplementary Table S1.

**Figure 4:**
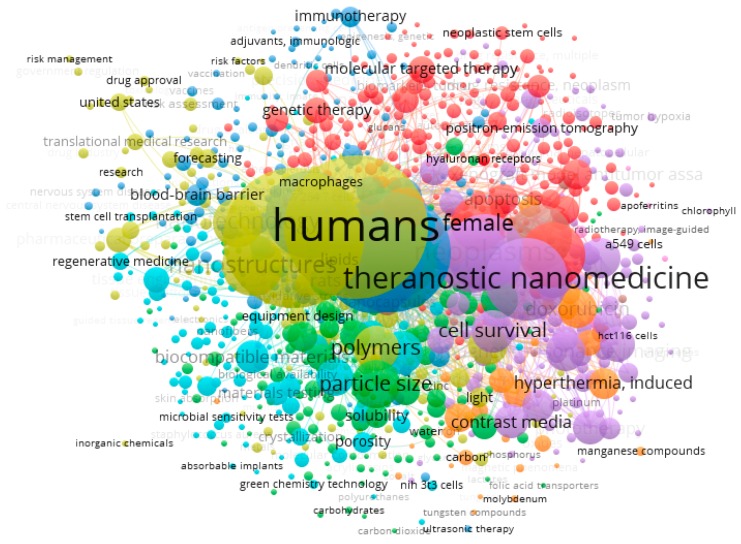
Topics addressed by investigations in the field of nanomedicine. Colors represent the different thematic clusters.

**Figure 5:**
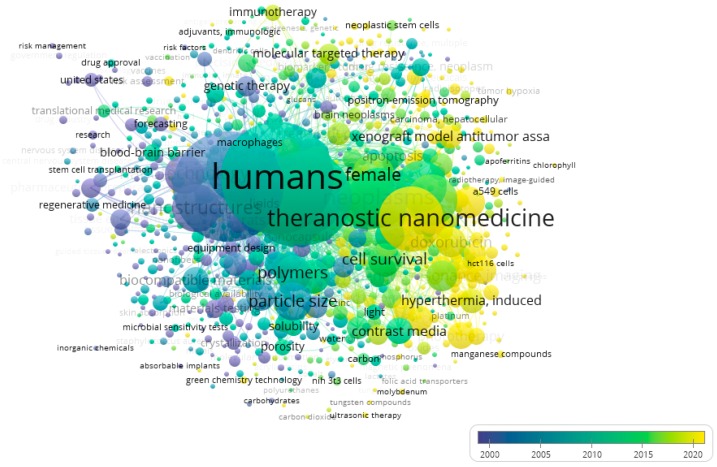
Topics addressed by investigations in the field of nanomedicine. Colors represent the fractions of articles published per year.

Topics are shown in Figure 4. Based on publication year, recent research has particularly focused on topics included in clusters 3 and 5 (Figure 5).

The countries with the highest percentages of articles in the field of nanomedicine were North America (38.3%) and Europe (35.1%) (Table 2). The different countries/regions are shown in Supplementary Figures S1–S5. Color code (green or gray) and proportion values (positive or negative) should be interpreted, taking into account that generated graphs are choropleth maps showing the proportion of results for a given search in each country with respect to all geo-tagged PubMed/MEDLINE research results. In other words, if the share of nanomedicine related articles/items for that country is higher (or lower) than the share of all articles produced in that country, the relative value will be positive (or negative) and the color code will be green (or gray). From the graphs, it can be seen that nanomedicine is a hot topic and nanomedicine-related research is generally over-represented with respect to other research topics in most countries, with few notable exceptions, such as Canada (Supplementary Figure S1), Spain, Denmark and Poland (Supplementary Figure S2), Nigeria (Supplementary Figure S3), Japan (Supplementary Figure S4), and Australia (Supplementary Figure S5).

## 4. Discussion

The present study showed that there is an increasing trend in publishing and performing research in the super-specialty of nanomedicine. Most productive countries were the USA and European countries, with China as emerging region. Hot topics in the last years were nano-diagnostics and nano-theranostics and clinical applications in the sub-fields of nano-oncology and nano-infectiology.

There are few scholarly investigations concerning the publishing trends related to the medical applications of nanotechnologies and nanosciences. Teles and collaborators [17] have focused on the potential promises of nanomedicine for the management and treatment of triple negative breast cancer (TNBC), mining the literature (Scopus database) from 2012 to 2017. Authors found that most articles concerned antineoplastic agents tested using in-vitro models, cell cultures, or animal models. A hot research topic was the “Michigan Cancer Foundation-7” (MCF-7) cell line [18]. Approximately less than one third of published articles were produced in the USA, as well as one third of received citations (28.36% and 27.61%, respectively). The journals “Biomaterials” and the “International Journal of Nanomedicine” published the highest number of investigations, with the USA and China having the highest number of scholarly items produced and cited, even though Singapore reported the highest mean number of citations per article.

Robert and colleagues [19] explored the publication trends of drug delivery from 1974 to 2015 mining the Science Citation Index Expanded database. Authors noted an exponential growth of publications in the period from 2004 to 2015, with countries like the USA, UK, Germany, Japan, Italy, France, and Canada mainly contributing to this increase. In recent years, regions such as China, India, and South Korea were particularly productive. Journals like the “Journal of Controlled Release”, “Advanced Drug Delivery Reviews”, and the “International Journal of Pharmaceutics” were the three top journals, publishing nearly one-fifth of the drug delivery research in the last years.

Ma et al. [20] reviewed the clinical applications of nano-enabled drug delivery systems in the field of nano-neurology (Alzheimer’s disease) and nano-oncology (brain cancer) and their evolutions, mining the PubMED/MEDLINE database. Dong and coworkers [21] mined the content of PubMed from 2002 to 2011 and analyzed 2543 articles published in over 50 scholarly journals from over 30 countries. The USA was the leading country, followed by Japan, Germany, France, China, and India. Articles focused on topics like nanoparticles, tumor biomarkers, and drug delivery. Szebeni [22] reviewed the literature on nano-psychiatry, finding that targeted pharmacotherapy enabled by nanocarriers (such as liposomes, micelles, polymer-conjugates, polymerosomes, dendrimers, aptamers and carbon nanotubes) represented the hottest research topic in the field, whereas lipid-polymer hybrid nanoparticles, next-generation core-shell nanostructures, as well as other bio-inspired nanoparticles appear to be promising nano-carriers in the field of nano-oncology [23,24], especially for cancers that are difficult to treat, like lung cancer [25].

Other existing articles are focused on specific journals, like “Theranostics” [26], or countries, such as Mexico [27].

To summarize, nanomedicine- and nanotechnology-based approaches appear to have an enormous potential, even though a gap between the wet-lab and clinical practice remains to be properly addressed. Moving nanomedicine forward “from the bench to the bedside” undoubtedly represents the future challenge [28]. Besides clinical translational development, other issues are biosafety, regulatory aspects, cost-effectiveness, as well as ethical implications emerging from the field of nanomedicine [29].

However, despite its strengths (robust methodology, high reproducibility), our investigation is not without any limitation. The major shortcoming is given by the search limited to only one database (even though PubMed/MEDLINE is the largest biomedical repository, with over 30 million articles, and the most commonly used database by researchers in the field). As such, further researches including other relevant repositories should be performed.

## 5. Conclusions

The present investigation demonstrated an increasing trend in publishing and doing research in the super-specialty of nanomedicine, with the USA and European countries as the most productive settings, and with China as an emerging region. Topics garnering recent interest from the academic communities were nano-diagnostics and nano-theranostics, as well as clinical applications in the sub-fields of nano-oncology and nano-infectiology. However, given the above-mentioned limitations further research in the field is warranted.

## Figures and Tables

**Table 1:** Number of clusters, number of items per cluster, and topic based on analysis of keywords and their co-occurrence.

Number of Cluster	Number of Items Per Cluster	Topic
First cluster	279	Molecular methods
Second cluster	235	Molecular biology, nano-characterization
Third cluster	185	Nano-diagnostics and nano-theranostics
Fourth cluster	174	Clinical applications (nano-oncology, nano-immunology and nano-vaccinology)
Fifth cluster	118	Clinical applications (nano-oncology and nano-infectiology)
Sixth cluster	10	Nanodrugs

**Table 2:** Percentage of articles in the field of nanomedicine per country regions.

Country Region	Percentage of Articles Related to Nanomedicine
North America	38.3%
Europe	35.1%
Asia	18.3%
Africa	5.4%
Oceania	2.3%
Central and South America	0.6%

## Supplementary Materials

The following are available online at https://www.mdpi.com/1010-660X/55/12/785/s1, Table S1: List of university institutions/organizations or research centers with more than 5 articles published in the field of nanomedicine, Figure S1: Publishing and research trends in the field of nanomedicine for North America, Figure S2: Publishing and research trends in the field of nanomedicine for Europe, Figure S3: Publishing and research trends in the field of nanomedicine for Africa, Figure S4: Publishing and research trends in the field of nanomedicine for Asia, Figure S5: Publishing and research trends in the field of nanomedicine for Oceania.
