# Supplementary material for: Nanomedicine: Insights from a Bibliometrics-Based Analysis of Emerging Publishing and Research Trends
Source: Medicina (Kaunas). 2019 Dec 15;55(12):785. doi: 10.3390/medicina55120785 (PMC6956084; doi:10.3390/medicina55120785)
Supplement: Supplementary file 1 [file medicina-55-00785-s001.pdf]

Nanomedicine: Insights from a Bibliometrics-Based Analysis of Emerging Publishing and Research Trends

Supplementary Table S1. List of university institutions/organizations or research centers with more than 5 articles published in the field of nano-medicine.

University Institution/Organization or Research Center	City	Country	Number of Documents
Center for Nanoscience and Nanotechnology, Tel Aviv University	Tel Aviv	Israel	5
Center of Excellence for Advanced Materials Research, King Abdulaziz University	Jeddah	Saudi Arabia	6
Department of Bioengineering, Graduate School of Engineering, The University of Tokyo	Tokyo	Japan	5
Department of Biomedical Engineering and Environmental Sciences, National Tsing Hua University	Hsinchu	Taiwan	5
Department of Biomedical Engineering, College of Engineering, Peking University	Beijing	China	13
Department of Biomedical Engineering, University of Southern California	Los Angeles	California, USA	5
Department of Chemical Engineering, Northeastern University	Boston	Massachusetts, USA	8
Department Of Pharmaceutical Sciences, University Of Nebraska Medical Center	Omaha	Nebraska, USA	5
Department of Pharmaceutics, College of Pharmacy, Third Military Medical University	Chongqing	China	5
Department of Pharmaceutics, Utrecht Institute for Pharmaceutical Sciences, Utrecht University	Utrecht	The Netherlands	5

Department of Pharmacology and Experimental Neuroscience, University Of Nebraska Medical Center	Omaha	Nebraska, USA	5
Drug Applied Research Center, Tabriz University of Medical Sciences	Tabriz	Iran	10
Immunology Research Center, Tabriz University of Medical Sciences	Tabriz	Iran	6
Jiangsu Key Laboratory of Translational Research and Therapy for Neuro-Psycho-Diseases, College of Pharmaceutical Sciences, Soochow University	Suzhou	China	5
Laboratory of Molecular Imaging and Nanomedicine, National Institute of Biomedical Imaging and Bioengineering, National Institutes of Health	Bethesda	Maryland, USA	22
Nanotechnology Research Center, Faculty of Pharmacy, Tehran University of Medical Sciences	Tehran	Iran	6
Pharmaceutical Research Center, Mashhad University of Medical Sciences	Mashhad	Iran	5
State Key Laboratory for Modification of Chemical Fibers and Polymer Materials, College of Materials Science and Engineering, Donghua University	Shanghai	China	7
State Key Laboratory of High Performance Ceramics and Superfine Microstructure, Shanghai Institute of Ceramics, Chinese Academy of Sciences	Shanghai	China	5

Université Catholique de Louvain, Louvain Drug Research Institute, Advanced Drug Delivery And Biomaterials	Brussels	Belgium	5
University of Chinese Academy of Sciences	Beijing	China	24

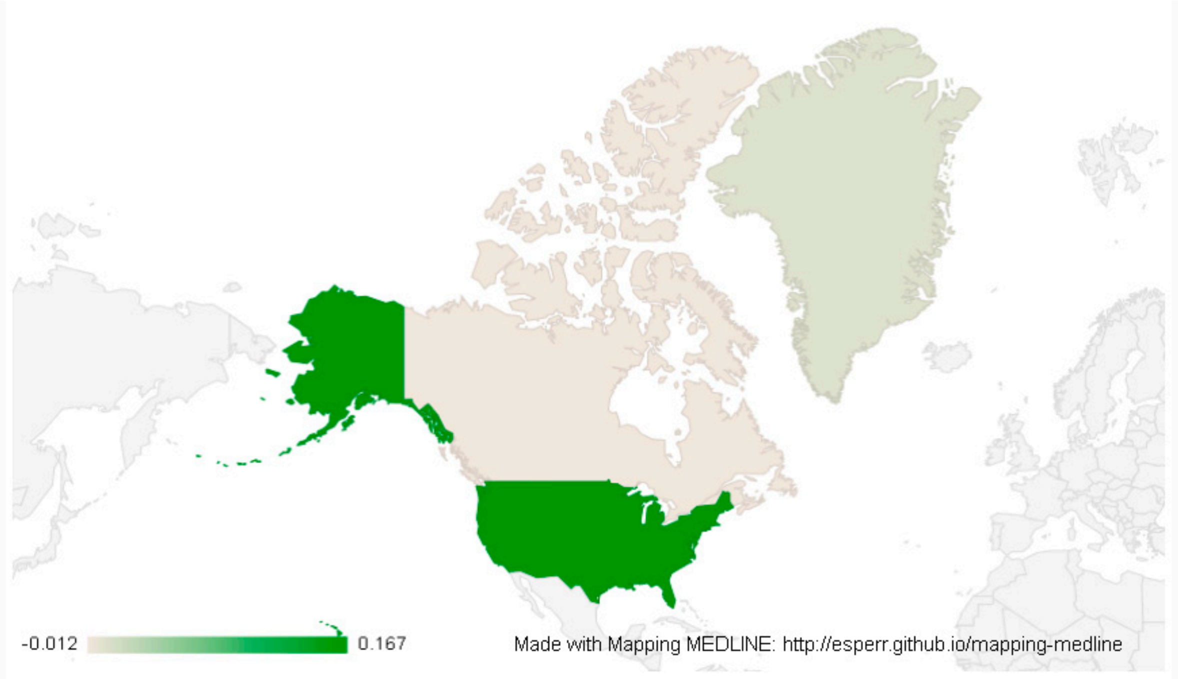

Supplementary Figure S1. Publishing and research trends in the field of nano-medicine for North America.

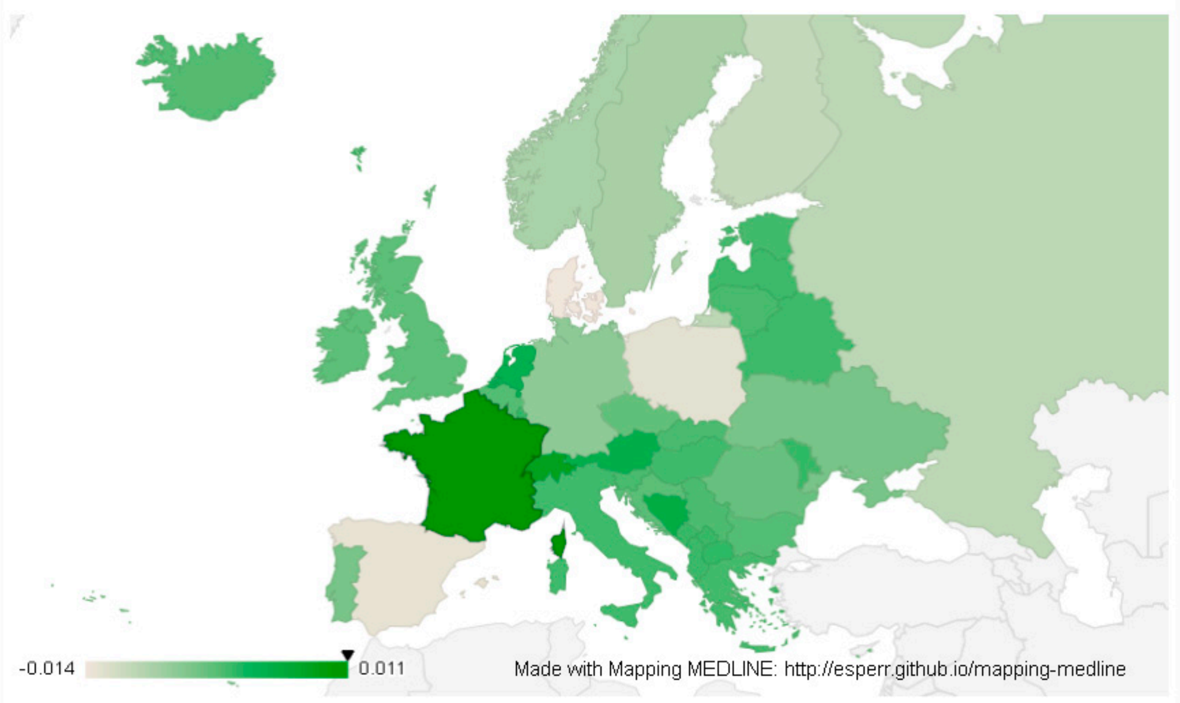

Supplementary Figure S2. Publishing and research trends in the field of nano-medicine for Europe.

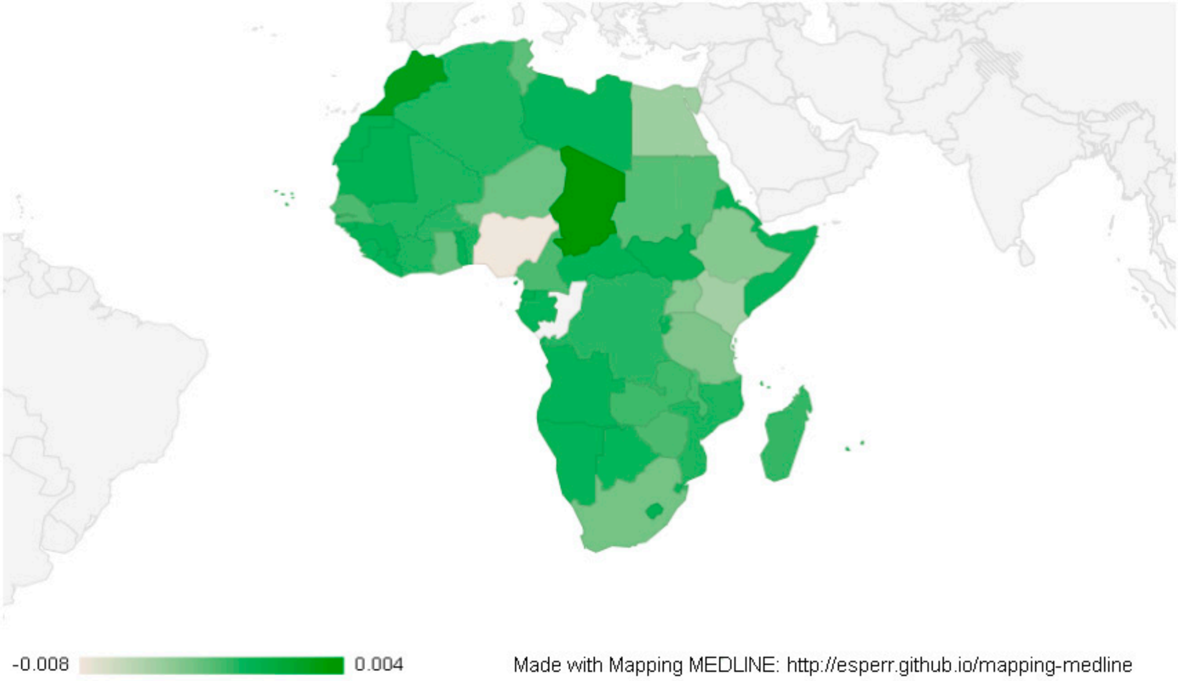

Supplementary Figure S3. Publishing and research trends in the field of nano-medicine for Africa.

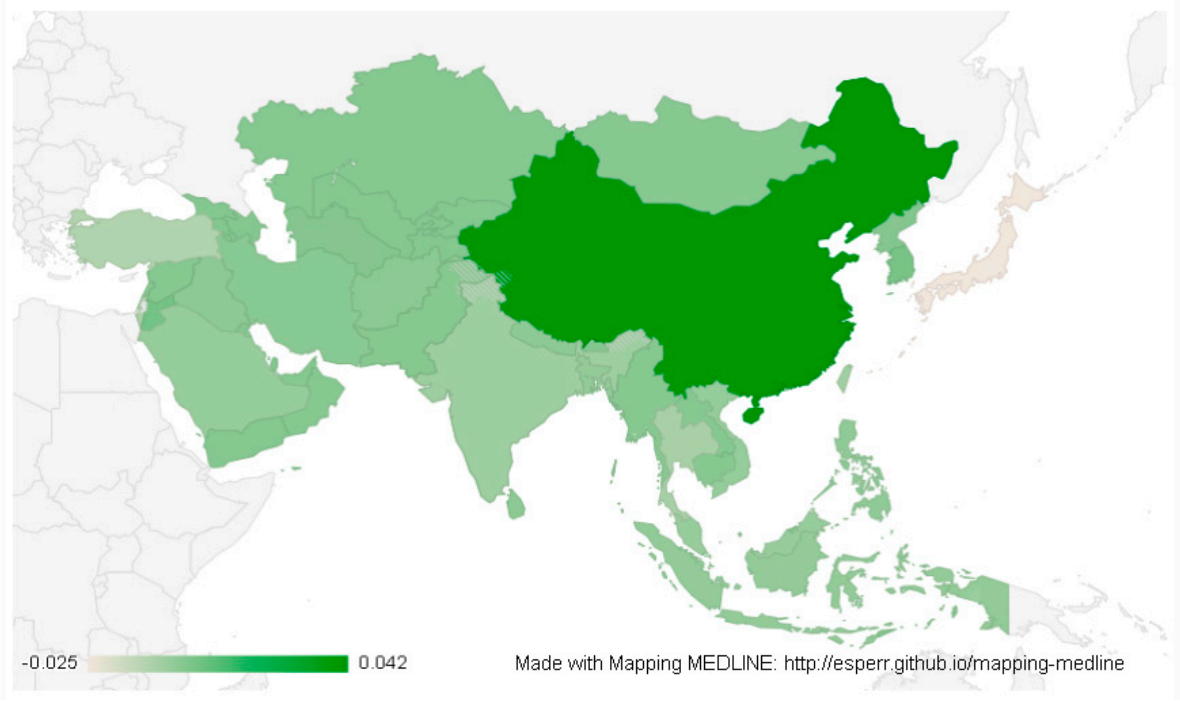

Supplementary Figure S4. Publishing and research trends in the field of nano-medicine for Asia.

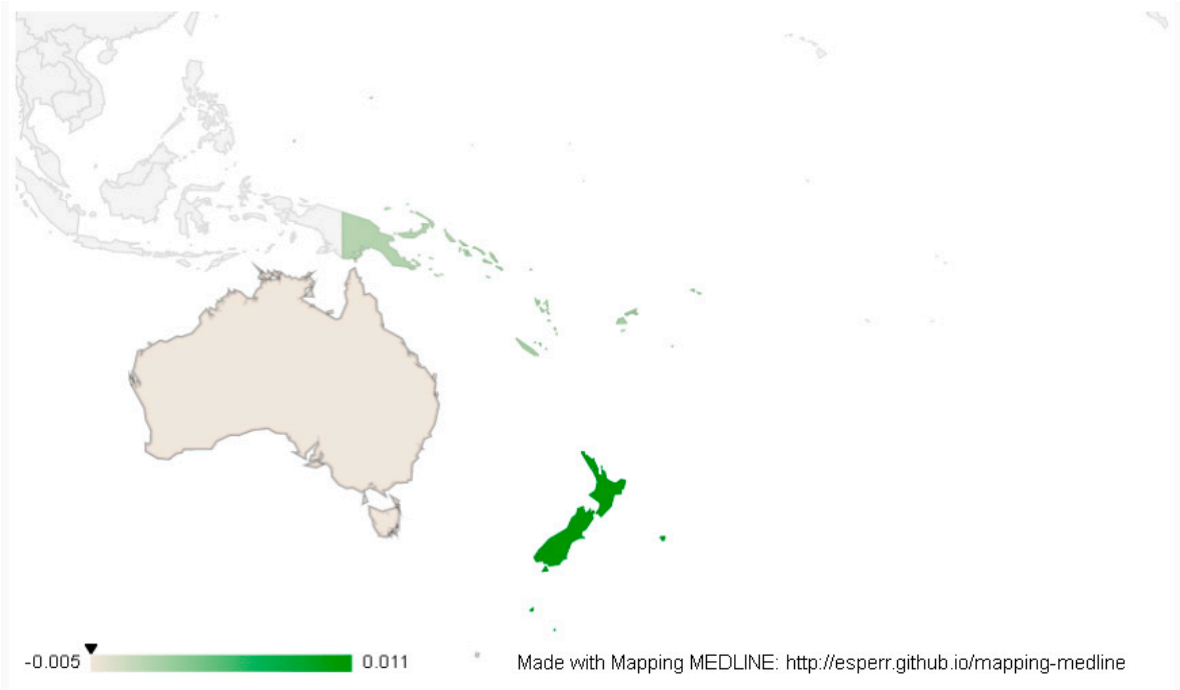

Supplementary Figure S5. Publishing and research trends in the field of nano-medicine for Oceania.
